# The Triple Jags of Dietary Fibers in Cereals: How Biotechnology Is **Longing** for High **Fiber** **Grains**

**DOI:** 10.3389/fpls.2021.745579

**Published:** 2021-09-14

**Authors:** Ermelinda Botticella, Daniel Valentin Savatin, Francesco Sestili

**Affiliations:** ^1^Institute of Sciences of Food Production (ISPA), National Research Council (CNR), Lecce, Italy; ^2^Department of Agriculture and Forest Sciences (DAFNE), University of Tuscia, Viterbo, Italy

**Keywords:** dietary fibers, cereals, arabinoxylans, β-glucans, resistant starch, crop biotechnologies, health

## Abstract

Cereals represent an important source of beneficial compounds for human health, such as macro- and micronutrients, vitamins, and bioactive molecules. Generally, the consumption of whole-grain products is associated with significant health benefits, due to the elevated amount of dietary fiber (DF). However, the consumption of whole-grain foods is still modest compared to more refined products. In this sense, it is worth focusing on the increase of DF fractions inside the inner compartment of the seed, the endosperm, which represents the main part of the derived flour. The main components of the grain fiber are arabinoxylan (AX), β-glucan (βG), and resistant starch (RS). These three components are differently distributed in grains, however, all of them are represented in the endosperm. AX and βG, classified as non-starch polysaccharides (NSP), are in cell walls, whereas, RS is in the endosperm, being a starch fraction. As the chemical structure of DFs influences their digestibility, the identification of key actors involved in their metabolism can pave the way to improve their function in human health. Here, we reviewed the main achievements of plant biotechnologies in DFs manipulation in cereals, highlighting new genetic targets to be exploited, and main issues to face to increase the potential of cereals in fighting malnutrition.

## Highlights

The application of modern biotechnology to crops is crucial to obtain cereals enriched in dietary fibers, essential bioactive compounds for human health.

## Introduction

**FIGURE 1:**
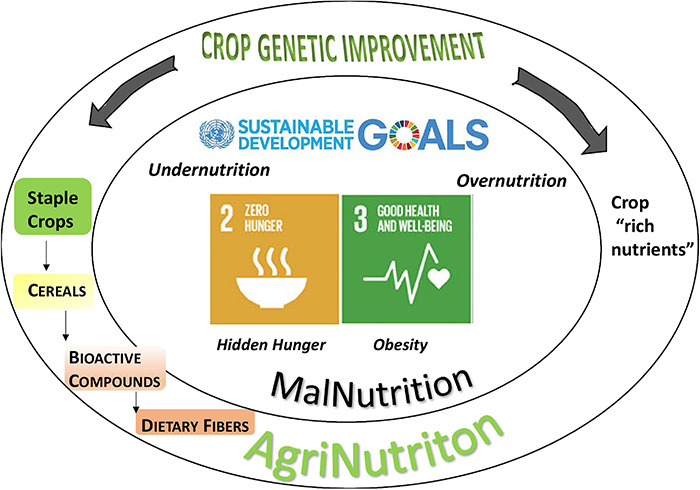
The role of cereals in the fight against malnutrition. The widespread consumption of cereals makes them ideal to deliver health benefits encountering the ask of the Sustainable Development Goals (SDGs) 2 and 3 for Zero Hunger. Dietary fibers are bioactive compounds playing an important role in human health: breeding to increase their content in cereal grains is a promising approach to malnutrition issue; in comparison to “crops rich nutrient,” cereals offer a more effective delivery in the diet.

Malnutrition is among the main issues in human health and involves three biggest concerns on the population diet: undernutrition (hunger), micronutrient deficiency (hidden hunger), and overnutrition (obesity). In this regard, nutrition research is called to meet the increasing demand for both staple crops and “nutrient-rich foods” (Zhou and Staatz, 2016). In some viewpoints, staple crops, primarily cereals, are neglected as “nutrient-poor foods” sourcing mostly dietary energy, therefore being responsible for overnutrition issues (Sanchez, 2020). Noteworthy, cereals play a key part in the human diet since they provide more than half of all calories consumed by humans, being a source of macro nutrients, carbohydrates and proteins, and significant amounts of bioactive compounds such as vitamins, minerals, and dietary fibers. The widespread consumption of major cereals makes them ideal to deliver benefits to the most of population due to consolidated diet styles: in this perspective, these crops are called to answer an urgent demand for healthiness by boosting the provision of essential bioactive compounds such as DFs (Poole et al., 2020; Figure 1). DFs have received plenty of definitions finely discussed elsewhere (Stephen et al., 2017), the most widely accepted being: “carbohydrate polymers with three or more monomeric units which are neither digested nor absorbed in the small intestine” (Commission Directive 2008/100/EC, 28 October 2018). Major DFs in cereals are βG and arabinoxylans, lignin, fructans, and resistant starch (Shewry et al., 2020).

Health aspects of DF span through many beneficial impacts on colonic function, short-term effects on glycemia, and regulation of blood cholesterol (Anderson et al., 2009) associated with the prevention of hard diseases connected to overnutrition, namely cardiovascular diseases (CVDs), diabetes II and some types of cancer.

These pathologies fall among non-communicable diseases (NCDs) and provoke death globally in 41 million people each year, equivalent to 71% of all deaths (WHO, 2021). DFs are amenable to mitigate major metabolic risk factors like overweight, obesity, hyperglycemia, raised blood pressure by plenty of physiological actions: increasing the viscosity of digesta in the small intestine; promoting prebiotic fermentation in the distal colon; accelerating the intestinal transit; sequestering of carcinogenic cells and cholesterol (Capuano, 2017). Recommendations for DF intake in adults in most countries (Europe and United States, Australia and New Zealand) are far away to be met: the recommended intake is in the order of 30 g/d, while the average intake accounts for 18 g/d in most countries (Stephen et al., 2017).

Cereal food accounts for the largest intake of fiber worldwide, providing from 32 to 48–49% in the United States and Europe respectively (Esteban et al., 2017; Stephen et al., 2017). For instance, the largest proportion of the RS comes from grain products.

That stated, the contribution of each cereal to the total DF diet intake varies greatly depending on several factors: the spread of cereals in the world diet, impact of food processing, distribution of fiber components across the seed layers.

Maize, rice, and wheat account for 89% of total cereal production worldwide, while barley, oat, and rye are categorized as “minor” or “specialty” grains.

In terms of human consumption, rice and wheat are the main crops, while maize production is primarily used in animal feed with only 15% of the grain used for food. Nevertheless, maize is a major staple in such areas as Africa and Latin America with more than 90% used for food (FAO, 2011). Barley is scarcely used for food production (about 6% of the total production), even if, due to its resilience, is still a staple crop in some areas of North Africa and the Near East (Zhou, 2010). Moreover, barley consumption is increasing in developed countries due to the growing awareness of its health properties. Other cereals, like oat and rye, are less important, as their impacts on the global diets are minor. However, rye, the richest in DF among cereals, plays an important role in the diet of several Nordic European countries, associated with relevant health benefits for the population (Jonsson et al., 2018).

Among major cereals, wheat grains contain 12% of DF, followed by maize (7.3%) and brown rice (3.4%); barley (*Hordeum vulgare*) has the highest content in DF (17%) (Prasadi and Joye, 2020). Minor cereals, such as oat and rye, contains 10.5 and up to 20% of total DF. However, DF intake in cereal foods strongly depends on the relationship between their distribution inside kernel layers and seed processing. Cereal grains are composed of several compartments: (i) the external layers (seed coat, pericarp, and aleurone), commonly defined as bran, are the richest in NSP and are removed by milling, whereas are stored in wholegrain products; (ii) the endosperm, mainly composed by starch and proteins, generally contains a lower amount of NSP as well as a trivial amount of resistant starch and represents the most of refined flour; (iii) the embryo, the vital compartment of the seed is removed by milling. If it is true that DFs are much higher in whole grain foods (11–15%), nonetheless consumer preference makes consumption of whole grains inadequate compared to more processed and refined foods. It derives that, beyond promoting education on the benefits of wholegrain foods to consumers, increasing the amount of DF in the endosperm has a great potential to enhance their intake in the diet. Therefore, the distribution of DFs across the seed layers differs widely among different cereals, with some species more amenable to increase the amount of DF in the endosperm.

Dietary fibers fractions majorly represented in the inner compartment of the seed are AXs, βGs, and RS. Arabinoxylans account for 70% of total DF in the wheat endosperm, while βGs are the predominant fiber in barley. In this frame, RS is advantageous for being in the endosperm. It is a starch fraction, whose physiological behavior retraces that of cell wall DFs: it results recalcitrant in amylases hydrolysis thus reducing the level of glucose release in the blood (low glycemic index) and is fermented by the gut microbiota promoting the release of small metabolites, the short chains fatty acids (SCFAs), beneficial for colon health (Regina et al., 2006). The consumption of foods enriched in RS can decrease glycemic and insulin responses and reduce the risk of developing type II diabetes mellitus, obesity, and cardiovascular diseases (Birt et al., 2013; Bird and Regina, 2018; Guo et al., 2020).

Resistant starch content in cereals depends on some major features, among which, the percentage of amylose in the reserve starch. Several studies revealed the existence of a correlation between the amylose content in the kernel and the amount of RS in flours and foods. High-amylose starches are now available in maize, rice, barley, and wheat. A rising number of studies are now evaluating possible beneficial effects on the health of enriched-resistant starch foods (Hogg et al., 2015; Schönhofen et al., 2017; Vetrani et al., 2018; Corrado et al., 2020; Sissons et al., 2020).

Arabinoxylan, βG, and RS, present at various levels in the endosperm, provide together a good target for crop genetic improvement focused on the enrichment of cereal flours in DF.

Although they share similar physiological behaviors, still, each class is associated with specific functionalities both at health and technological levels: their different chemical nature deeply conditions short-chain fatty acids (SCFAs) production and gut microbiota composition (Tiwari et al., 2019); as well as their differences in solubility profiles greatly affect both food digestibility and its technological properties (Buttriss and Stokes, 2008; Elleuch et al., 2011). Moreover, the amount and percentages of AX, βG, and RS are variable among the different cereals. All considered it seems to us, that to answer to the demand for cereal food enriched in DFs, it is of interest to consider all three groups of these DFs together with the following aims: ideation of strategies able to boost the content of more than one class at a time; finding a good compromise between healthy and technological properties; choosing the best DF target in consideration of the cereal species and the main end-products properties (Stephen et al., 2017).

Dietary fiber enrichment in cereal by genetic approaches relates to the investigation of the metabolic pathways and genetic determinants responsible for their accumulation and structure-functionality relationship in all major species.

An overall analysis of the main actors playing a key role at metabolic and gene levels can help to highlight crucial relationships between the three carbohydrates, useful for appropriate modulation of DF amount in the endosperm.

In this review, we revised studies centered on three main DF classes: βGs, and AXs, and RS. The biosynthetic pathways and key enzymes involved are discussed, highlighting putative main regulators to be considered for future breeding purposes; in addition, studies until so far performed by involving classical and modern crop genetic improvement tools, aiming to influence the amount or modulate the structure of the three DFs, are revised.

Lastly, we highlight novel questions to be addressed and possible targets of interest to be exploited to obtain new cereals biofortified in AXs, βGs, and RS.

## DFs in Cereal Grain

Arabinoxylan, βG, along with cellulose, and the non-carbohydrate component, lignin, are the predominant cell wall polysaccharides in cereals. They occur in different proportions depending on the species and tissue type. Wheat along with maize, is rich in AX, whereas barley and oat contain a modest amount of AX and a high level of βG. AX from wheat and βG from barley and oat are mostly soluble, whereas AX from maize is mainly insoluble (Knudsen, 2014). Solubility impacts their physiological properties with soluble DF (SDF) playing the most of healthy functions (Prasadi and Joye, 2020).

Arabinoxylan and βG are the main components of the cell walls both in the endosperm and aleurone tissue, while in the pericarp there is a large presence of other cell wall constituents.

In barley, βGs are evenly distributed in the sub aleurone layers and endosperm where represent the major endosperm cell wall component (75% of the total cell wall material) (Fincher and Stone, 1986). AXs are the major components of the cell wall in the wheat endosperm reaching up to 67%, whereas the βG content is about 27% (Supplementary Table 1 and Supplementary Figure 1) (Fincher and Stone, 1986). The remaining fraction is mainly constituted by glucomannan and cellulose (Bacic and Stone, 1981). Differently, the third component, the resistant starch, is normally present at low levels in most cereals. Based on the prominence of AXs in wheat endosperm and βG in barley one, these DFs will be mainly discussed in these two cereals. For RS, the discussion will be focused on wheat, rice, maize, and barley, as they were the object of several breeding programs focused on the achievement of genotypes with an elevated amount of amylose and RS.

## Arabinoxylans, a Complex Structure

Arabinoxylans consist of a linear backbone of β-1,4-linked D-xylose residues with frequent substitutions with L-arabinose. In the case of mono-substitutions and di-substitutions, L-arabinose is inserted in the O-2 or/and O-3 positions (Courtin and Delcour, 2002). In addition, ferulic acid (FA) or coumaric acid (pCA) can be ester-linked to the O-5 of arabinose (Harholt et al., 2010). FA and pCA are the most abundant phenolic acids in cereals and play a crucial role in the regulation of the properties of the cell wall as cellular interaction and rigidity (Hassan and Burton, 2018). Indeed, the presence of FA esters favors cross-linkages to other FA esters or lignin; these interactions are important for the structure of the cell wall matrix (Grabber et al., 1995; Vogel, 2008). AXs structure shows a high variability inter and intraspecies and among the different seed layers with a great diversity of side chains and composition; although less frequent, single units of α-d-glucuronic acid and 4-*O*-methyl-glucuronic acid can be attached to the backbone at O-2 and O-3 positions, as well as the association of xylose and galactose residues with arabinose, are also found as short sugar units. Notably, AXs of outer layers of the seed, pericarp, and aleurone, where the cell wall material contributes for 40–60% of the dry weight, show a more complex structure with several substituents such as pCA and a higher percentage of FA. Indeed, ester-linked pCA is not detected in pure starchy endosperm tissue dissected from wheat grain (Barron et al., 2007).

Empirically, AXs have been classified in water-extractable (WE-AX) and water-unextractable (WU-AX) fractions. This latter can be extracted using an alkaline solution able to break ester linkages (Courtin and Delcour, 2002; Kiszonas et al., 2013). Differently from WU-AXs, WE-AXs are not retained in the cell walls through covalent and non-covalent interactions with other wall components (proteins, cellulose, and lignin), but they are weakly bound on the cell wall surface. Their water-extractable nature has been not fully elucidated: Courtin and Delcour (2002) hypothesized incomplete cross-linking with other wall components in the kernel. In wheat kernel, WE-AXs constitute about 25–30% of the total AXs (Saulnier et al., 2007). The ratio between arabinose and xylose (A/X) is associated with the polysaccharide solubility; indeed, the presence of arabinose side chains limits the aggregation between the linear xylose chains thus increasing the interaction with the solvent. However, the arabinose is susceptible to esterification by hydroxycinnamic acids, mainly FA, that promotes the crosslinking (di-tri ferulates) between the xylan chains, reducing solvent accessibility. Diferulate cross-linking affects the physio-chemical properties (notably solubility and viscosity) of these polymers (Shewry et al., 2020). The solubility of AXs affects their physiological properties and impacts positively on human health; this correlation is far to be evenly comprised and current knowledge has been recently and widely reviewed in Wang et al. (2020a). Among the three major cereals, wheat is richer in water-soluble AXs (WE-AXs) only found in the cell wall of starchy endosperm.

## Arabinoxylan Biosynthesis

**FIGURE 2:**
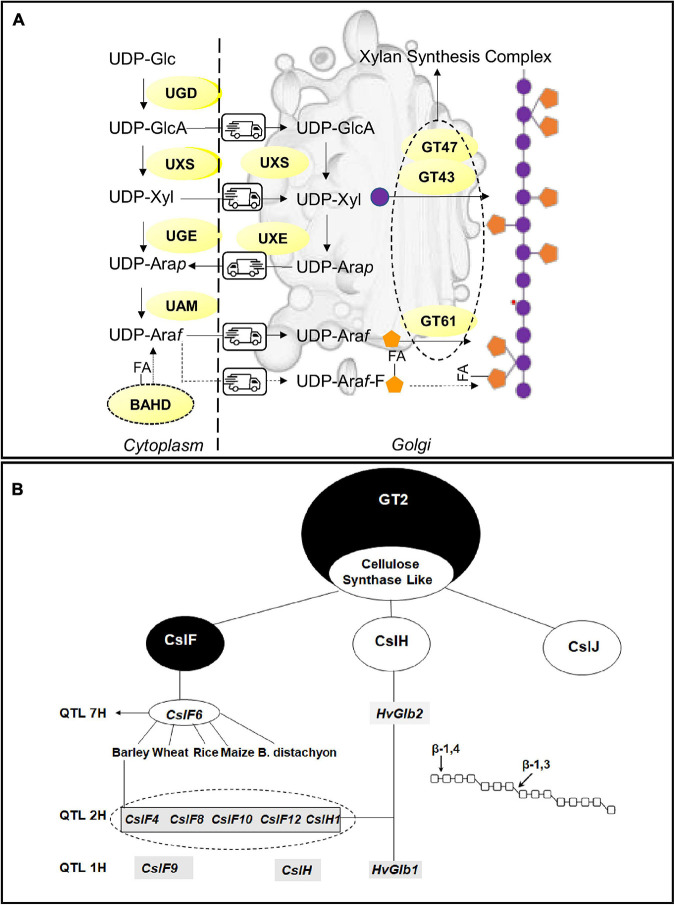
Biosynthetic pathways and key enzymes for **(A)** arabinoxylans and **(B)** β-glucans. **(A)** Schematic representation of arabinoxylan synthesis involving the cytoplasm and Golgi apparatus; nucleotide sugars: Xyl, Xylose; Ara, Arabinose; GlcA, Glucuronic acid; UDP-L-Arap, UDP-L-arabinopyranose; UDP-L-Araf, UDP-L-arabinofuranose. UDP-sugars converting enzymes UAM, UDP-Ara mutase; UGD, UDP-Glc 6-dehydrogenase; UGE, UDP-glucose 4-epimerase; UXE, UDP-Xyl 4-epimerase; UXS, UDP-Xyl synthase; BAHD acyl transferase FA, Ferulic acid. GT47, GT43, GT61 Glycosytransferases. **(B)** Key Enzymes involved in β-glucan synthesis in cereals. The figure shows the main QTLs and associated candidate genes for β-glucan synthesis identified in cereals. The dotted circle underlines the cluster of *CslF* plus *CslH1* genes identified in barley.

The build-up of the AX backbone occurs in the Golgi in a deep dialog of metabolites with the cytosol, mediated by several membrane channels, the (UDP)-sugar transporters. The monomers of the polysaccharide, xylose and arabinose are synthesized both in the cytoplasm and in the Golgi due to the presence of multiple isoforms of the synthetic enzymes. Most of the investigation on AX biosynthesis in cereals focalized on the step of glucan chain construction; nonetheless, the knowledge of the mechanism of xylose and arabinose synthesis can provide new opportunities for the modification of AX fine structure in cereals (Figure 2A).

### Crosstalk of Nucleoside Diphosphate (NDP) Sugar Precursors Between Cytosol and Golgi

The synthesis of all xylans starts from their precursors, the UDP-sugars, (di)phosphonucleotide-activated sugars, synthesized between the cytosol and Golgi apparatus. The UDP-D-xylose (UDP-Xyl), the monomer of the xylan backbone, is synthesized by the UDP-Xyl synthase (UXS), whose isoforms are localized both in the cytosol and Golgi apparatus, through the decarboxylation of UDP-D-glucuronic acid (UDP- GlcA). Reverse genetic approaches, targeting the genes coding for the UDP-Xyl transporters (UXT), demonstrated that cytosolic UXS isoforms, and not the Golgi-located ones, are essential for the synthesis of xylan chains (Zhao et al., 2018). Arabinose, the major substituent of the xylan chain, is obtained from the conversion of (UDP)-xylose in (UDP)-Arabinopyranose (Ara*p*) by a UDP arabinose epimerase (UXE). There are two and three UXE isoforms in rice and barley respectively (Seifert, 2018). It has been reported that the functionality of Golgi-UXS is essential to maintain a normal arabinose content in the cell wall (Saez-Aguayo et al., 2017). Once synthesized in the Golgi, Ara*p* is channeled back to the cytosol where the UAM complex (UDP-arabinopyranose mutase) interconverts UDP-Ara*p* to UDP-Arabinose furanose (Ara*f*); then, Ara*f* is taken back to the Golgi where it will be incorporated in AX structure. The other two suppliers of arabinose in the cells are the fueling caused by the carbohydrate breakdown running in the cytosol and the cytosolic UDP-Xyl conversion operated by the bifunctional enzyme UGE l (UXE/UDP-glucose 4-epimerase) (Kotake et al., 2009).

### Feruloylation

Hydroxycinnamic acid derivatives are the AXs’ unique feature. Ferulic, dehydrodiferulic, p-coumaric, and sinapic acids esterify the 5-OH of arabinosyl residues. These ferulic acid groups also form covalent bonds to other molecules in the cell wall, such as proteins, lignins, and other glucans (Saulnier et al., 2007). It has previously been suggested that the dominant characteristic determining AX solubility is the amount of diFA (Saulnier et al., 2007). Ferulic substituents form oxidatively-linked dimers and oligomers with cell wall polymers that result in a covalently linked network within the cell wall. Members of the “Mitchell clade” within the BAHD acyltransferase superfamily are involved in FA and pCA esterification of xylan in monocot cell walls. BAHD acyltransferases presumably transfer FA to an intermediate, such as UDP-Ara*f*, which is then transported into the Golgi and transferred onto the xylan chain by unknown proteins (Bartley et al., 2013).

### Synthesis of the Xylan Backbone and Araf Decoration

The building up of the AX structure implicates the cooperation of several enzymes belonging to two major families: the Glycosyltransferases (GTs) and Glycosyl hydrolases (GHs). Several subclasses of GT families exist, and the specificity of the role played by the single protein members is wide far from being elucidated. In addition, it is yet to be established if the biosynthesis of AXs in cereals involves a terminal oligosaccharide at the reducing end which might act as a primer or terminator (York and O’Neill, 2008). GTs transfer the sugar-activated precursors (NDP-sugars) onto a specific acceptor catalyzing the formation of glycosidic bonds. The model proposed for AX synthesis theorizes the existence of a multi-enzyme complex, the xylan synthase complex, composed of at least three members of the two different GT clades. Proteins coded by the three genes *IRX9*, *IRX10* (*GT43*), and *IRX14* (*GT47*) cooperate to the synthesis of the xylan chains in the Golgi apparatus. IRX9 was supposed to play a structural role as its lack of an aminoacidic motif essential for the synthetic activity (Urbanowicz et al., 2014). A hypothesis has been advanced about the involvement of unidentified members of the processive GT2 family in the biosynthesis of (1,4)-β-xylans essential for the processivity of the synthetic process (Bulone et al., 2019).

Enzymes involved in the decoration of the AX backbone with arabinosyl and xylosyl sidechains are members of the GT61 family. Two xylan arabinosyl transferases (XATs) in wheat (TaXAT1, TaXAT2) and rice (OsXAT2, OsXAT3) have been characterized both natively and in heterologous systems (Anders et al., 2012; Zhong et al., 2018). Other GT61 members can transfer xylosyl side chains on the xylan backbone: in rice, xylosyl-arabinosyl substitution by xylan-xylosyltransferase (OsXAX1) mediates the addition of xylose to arabinose units while rice xylan-xylosyltransferase 1 (OsXYXT1) adds xylose sidechains to the xylan backbone (Xyl*p*-1,2-b-Xyl*p*) (Zhong et al., 2018). Xylan-specific arabinosyl-transferase activities of GT61 enzymes in grasses provide new targets for the modification of xylan structure. Members of glycosyl hydrolases have been often associated with AX synthesis; it is postulated that they play a role in the modeling of AX fine structure recalling what happens for starch with debranching enzyme (Fincher, 2009).

### β-Glucans: A Simpler Polysaccharide

Within cereals, barley grains are the richest source of βGs with a values range of 2.5–11.5% of its dry weight, while wheat and maize have lower concentrations of the polysaccharide (Izydorczyk and Dexter, 2008; Supplementary Table 1). βGs from different sources and tissues present differences in their structure and properties: in barley, βGs from the endosperm have been reported to be 20% soluble in H_2_O at 40°C, while in wheat they are completely insoluble.

βGs present a simple primary structure composed of glucose units linked by both β-1,3 and β-1,4 glycosidic bonds. Specifically, 1,4-linked oligosaccharides composed of three (β-cellotriosyl) and four (β-cellotetraosyl-) glucose residues are linked by a single (1,3)-β-linkages. The ratio between (1,4)-β-glucosyl residues and (1,3)-β-glucosyl residues is approximately 3:1 with a wide variation across different species and tissues (Burton and Fincher, 2009). The coexistence of β-1,3 and β-1,4 linkages determines an asymmetry in the conformation of the polysaccharide that prevents the aggregation of the glucan chains increasing the flexibility and the solubility. Hence, the viscosity properties of βGs, associated with their healthy properties, are directly connected with its chemical structure: the ratio between β-cellotriosyl and β-cellotetraosyl (DP3/DP4) residues is a useful predictor of the polysaccharide solubility, where very high or very low ratios indicate low solubility, while ratios around 1.0:1–2.5:1 predict relatively higher solubility (Burton et al., 2010). The manipulation of the distribution of β-1,3 linkages interspaced across β-1,4 linked oligosaccharides represents a main target for the modulations of βGs solubility in cereals. Notably, longer blocks of (1,4)-β-glucosyl residues, up to 12 adjacent, have been found in cereal bran (Burton et al., 2010).

### β-Glucan Biosynthesis

In cereals, (1,3,1,4)-βGs are synthesized by enzymes belonging to the Cellulose Synthase Like Superfamily, within the large glycosyltransferase GT2 family (Bulone et al., 2019), specifically belonging to three groups specific for grasses code by the three genes *CslF*, *CslH*, and *CslJ* (Kumar et al., 2016; Figure 2B). *CslF6* gene, identified as the key candidate for the synthesis of (1,3;1,4)-βG in the barley endosperm (Burton et al., 2011), has been also studied in wheat, rice, maize, and *B. distachyon* (Nemeth et al., 2010; Coomey et al., 2020). In barley, the comparison of CslF6 with other members of the same clade identified an insertion of 55-amino acid residues essential for the amount and fine structure of the (1,3;1,4)-βG (Schreiber et al., 2014). Dimitroff et al. (2016) were able to identify regions of the enzyme that are important for overall (1,3;1,4)-βG synthesis and for defining the DP3:DP4 ratio of the polysaccharide chain. In detail, the N-terminal region of the CslF6 protein in barley, maize, and sorghum influences total βGs synthesis activity and the C-terminal region appears to influence the ratio of DP3/DP4 linkages.

These findings are of utmost importance for the manipulation and reprogramming of βG structure in cereals. Other enzymes belonging to *Csl* family are supposed to play a major role such as CslF9 (Burton and Fincher, 2009); recently, the targeting by genome-editing of several members of CslF and CslH clades in barley highlighted differences in βGs content only for the CslF6 knockout mutants (Garcia-Gimenez et al., 2020). CslH is supposed to take part in the remodeling of the polysaccharide, thus contributing to the definition of its fine structure.

Differences among cereal species have been detected concerning the location of βGs synthesis: Golgi-localized synthesis has been suggested for maize, whereas it occurs at the plasma membrane in barley and wheat (Bulone et al., 2019). Here, the synthase enzyme shows an intracellular or intra-organellar active site from which the nascent polysaccharide is extruded to the opposite side of the membrane through a pore formed from six transmembrane α-helices (Bulone et al., 2019).

## Resistant Starch in Cereal Grain

Among the five classes of RSs, RS type 3 is identified as “retrograded amylose.” Briefly, starch is overall composed of two distinct populations of α-glucans differing in the degree of polymerization (DP) and level of branching: amylose is a linear chain of α,1-4 linked glucose molecule and rare branches, with DP ranging from 10^2^–10^4^, while amylopectin is extremely branched due to a high level of α-1,6 side chains (1:12/15 glucose units) and shows a higher degree of polymerization (DP 10^4^–10^6^) (Zeeman et al., 2010). In cereals, the ratio between the two glucan polymers is mostly around 1:3 (amylose/amylopectin). The modulation of this ratio deeply affects physic-chemical properties of starch, influencing the starch gelatinization, solubility, retrogradation, and end-uses (Blazek and Copeland, 2008). Starch is organized in semi-crystalline structures, called granules, in which amylopectin chains clustered *via* hydrogen bonds forming double-helical structures distributed through layers in a hierarchical order (Buléon et al., 1998; Pérez and Bertoft, 2010). Amylopectin, with its high levels of ramification, is the main responsible for the ordered and crystalline feature of the starch granules. Branching triggers an opened kind-structure with void spaces easily hosting solvent molecules and hydrolytic enzymes: amylopectin is quite soluble in water and easily broken down in glucose units by digestive amylases. Quite the opposite, the linear chains of amylose tightly interact between them, deriving in a refractory arrangement not prone to be solubilized neither to be enzymatically processed: amylose properties reflect in “resistant starch” behavior, so-called since it withstands the enzyme action in the stomach and small intestine promoting lowering of glucose level in the blood after a meal (Sharma et al., 2008; Birt et al., 2013).

## Starch Biosynthesis

The starch precursor, ADP-glucose, is synthesized mostly in the cytosol by ADP glucose-pyrophosphorylase: glucose-1P reacts with ATP to generate ADP-glucose and PPi; afterward, ADP-glucose is transferred to the plastid by specific transporters.

**FIGURE 3:**
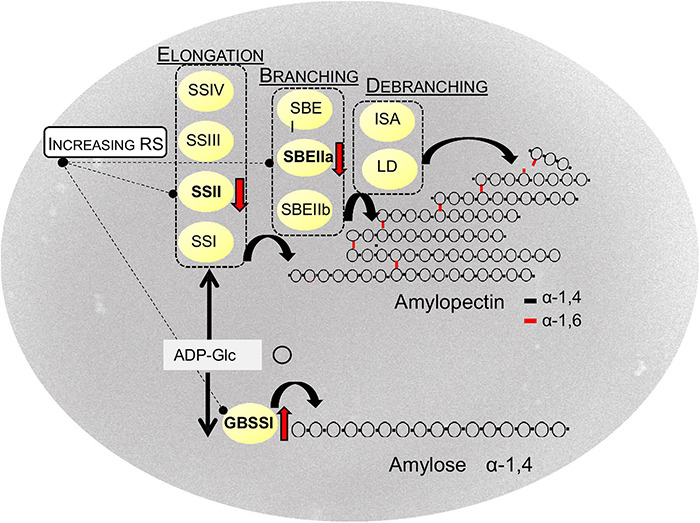
Biosynthesis of (resistant) starch in cereals. SS, starch synthases; SBE, starch branching enzymes; ISA, isoamylases; LD, limit dextrinase. GBSSI Granule Bound Starch Synthase, ADP-Glc: ADP-glucose. Red arrows indicate the main targets to increase RS.

Starch molecules are the product of a binary mechanism administered by an enzymatic complex including elongating, branching, and debranching enzymes (Figure 3). Granule Bound Starch synthase I (GBSSI) catalyzes the α-1,4 linkage between glucose units and is mostly associated with the synthesis of amylose molecules (James et al., 2003; Jeon et al., 2010). To be exact, a few reports have reported the implication of this enzyme also in the production of extra-long amylopectin chains (DP 300–500) (Hanashiro et al., 2008; Crofts et al., 2018). Amylose synthesis is most likely primed by short malto-oligosaccharides (MOSs) (DP 2–7) derived by various sources, processively elongated by GBSSI. Elongated MOSs with DP > 7, constrained into the granule, finally become long amylose chains (Denyer et al., 2001). Among the starch synthases, GBSSI takes on several exceptions: it is deficient in a Carbohydrate-Binding Module (CBM) in its structure; it is internalized within the granule and acts through a processive mode (Hebelstrup et al., 2017; Zhong et al., 2020). Putting together these considerations have helped to theorize that GBSSI acts in the interior of the granule where amylose remains protected by hydrolytic enzymes of the stroma (Seung, 2020); moreover, it may be that, in the absence of the amylopectin-environment, amylose would arrange in an insoluble compound, thus hindering its synthesis. The absence of a CBM module in the GBSSI structure is ascribable to its processive mode of action; a stable interaction between the nascent polymers and the enzyme would not fit with the inter-mobility of the two molecules. Recently, it has been found that the initial localization of GBSSI to the granule is mediated by specific proteins among which, the Protein Targeting to Starch 1 (PTST1) (Seung et al., 2015). In many species the impairing of PTST1 has been associated with low amylose content; in rice, CRISPR/Cas9 generated knocked mutants showing little effects on amylose content in the endosperm (Wang et al., 2020b); differently, starch synthesis was completely abolished in PTST1 knockout barley mutants generated through CRISPR/Cas9 technology (Zhong et al., 2019). Further studies are necessary to better explain the role of these proteins in cereals (Seung, 2020).

Amylopectin synthesis precedes the formation of amylose. It derives from the action of different isoforms of three classes of enzymes: starch synthases (SS), starch branching (SBE), and debranching enzymes (DBE). Four starch synthases (SSI, SSIIa, SSIII, and SSIV) have been characterized and related to the elongation of chains with different DP (Morell et al., 2001); among them, SSIIa elongates medium DP-chains and results essential for starch synthesis. Three isoforms for branching enzymes (SBEI, SBEIIa, SBEIIb) have been extensively characterized: SBEII has two isoforms in grasses with alternative distribution among granule and plastid stroma and results essential for the maintenance of branching pattern in amylopectin (Tetlow and Emes, 2014). SBEI is believed to play a helping role; it has been proposed to be involved in the branching of extra-long chains. Debranching enzymes cleave α-1,6 bonds and result essential for the crystalline structure of the granule: *sugary1* mutants in maize, rice, barley, and wheat, lacking isoamylase (ISA) activity, produce, instead of the starch granules, a water-soluble polyglucan structurally similar to glycogen (Pan and Nelson, 1984; Nakamura et al., 1996; Burton et al., 2002; Sestili et al., 2016). Moreover, branches trimmed by DBE enzymes are a source of malto-oligosaccharides, the substrate for starch synthases, or specific classes of enzymes, such as starch phosphorylase and disproportionating-enzyme involved in granule initiation (Myers et al., 2000).

### Transcriptional Regulation of Starch Biosynthesis

Several transcription factors (TFs) regulating starch synthesis have been reported in cereals. Most of them have been identified by co-expression analysis of starch synthetic genes that have largely increased thanks to NGS-based gene expression studies. Detailed analysis of the mode of action for each of these regulators has been reported: overall, it appears a coordinated mechanism in which each TF regulates the expression of many related starch genes. In barley *HvSUSIBA2*, involved in sucrose-mediated control of starch synthesis, directly interacts with *ISA* and *AGPase* genes (Sun et al., 2003). TFs of the bZIP family such as MYB, NAC (for NAM, ATAF and CUC) or AP2/EREBP families have been associated to the regulation of starch related genes (López-González et al., 2019).

OsbZIP58, a basic leucine zipper transcription factor, regulates positively the expression of *OsAGPL3*, *OsWx*, *OsSSIIa*, *OsSBE1*, *OsBEIIb*, and *OsISA*2 in rice (Wang et al., 2013); OsbZIP58 null mutants highlighted an abnormal seed morphology with reduced contents of total starch and amylose. Differently, OsRSR1, an APETALA2/ethylene-responsive element-binding protein family TF, acts as a negative regulator of starch gene expressions in rice seeds (Fu and Xue, 2010): it’s silencing increased amylose content, seed size, and yield; on the opposite, its overexpression repressed the expression of starch synthetic genes. In maize, many TFs have been associated with the regulation of key genes involved in starch synthesis (ZmABI4, ZmbZIP91r ZmMYB14 ZmDof3 ZmNAC128, ZmNAC36, and ZmNAC130) (López-González et al., 2019). ZmMYB14 also regulated the expression of the *Brittle 1* (*BT1*) gene, which encodes an ADP-glucose-transporter crucial for starch synthesis. Ethylene signaling has recently been linked in the transcriptional control of starch synthesis in rice, involving the ethylene receptor ETR and the AP2/EREBP family transcription factor (López-González et al., 2019). In wheat and rice endosperms the transcription factor TaNAC019-A1 negatively regulates starch synthesis. Its overexpression reduced significantly starch content, kernel weight, and kernel width (Liu et al., 2020). Recently, Song et al. (2020) identified a transcriptional activator of starch synthesis (TabZIP28) in wheat.

## Biotechnological Resources for Dietary Fiber in Cereals

**FIGURE 4:**
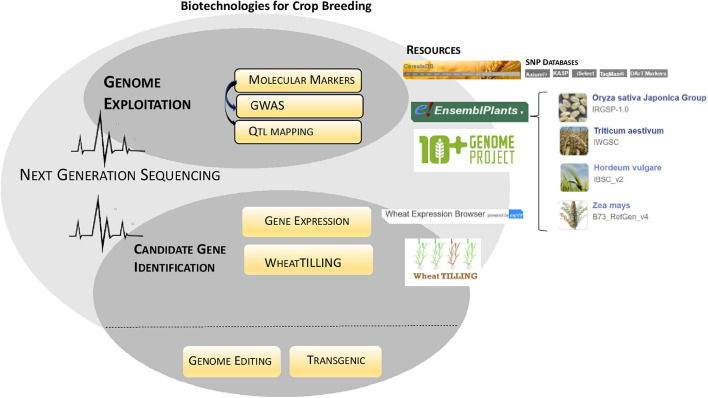
Biotechnologies for crop breeding. The figure summarizes the role played by advanced biotechnologies in crop breeding indicating novel resources now available (Resources). NGS has accelerated both genome exploitation and candidate gene identification by boosting molecular markers research (GWAS/QTL mapping) and transcriptomic/genetic variability studies, respectively.

In the era of genome editing, biotechnology promises an ultra-fine modulation of the genomes without introduction of foreign DNA. The inside potential of this technology is limitless, and much progress has been gained a few times. Single gene targeting has quickly been overcome by multiple loci manipulation through polycistronic designs, thus encountering the need to piece together multiple traits at one time (Camerlengo et al., 2020). Nowadays, we can edit genes precisely “correcting” even one single amino acid or the whole protein sequence (Wada et al., 2020). If this is true, that CRISPR-Cas can afford the challenge to precisely plan gene editing, at now, it is overall meant for reverse genetics purposes aiming at generating “loss of function” mutations in the genes of interest. The Non-Homologous End Joining mechanism, the most efficient in plants, repairs specific cuts run by Cas enzyme, generating small *in-del* in the sequence that prevents the gene functionality (Schmidt et al., 2019). Notably, in rice a collection of loss of function mutants spanning the genome has been generated, converting CRISPR-Cas into a high-throughput tool for mutagenesis (Lu et al., 2017). This approach is of interest to generate further genetic variability essential in plant genetic improvement (Bigini et al., 2021); in the coming years, the advantages of a CRISPR-Cas induced mutagenesis would be investigated in comparison with other well-established resources such as Targeting Induced Local Lesions IN Genomes (TILLING) (McCallum et al., 2000). Although CRISPR-Cas has revolutionary perspectives, it cannot be done without more classical resources that, over the decades, have allowed to exploit and tag the genome of the organisms associating DNA features to the phenotype: molecular markers have evolved incessantly assuming many forms that, the in last decade, have enormously taken advantage by high-throughput sequencing technologies (Figure 4). Considered that in plants, the most are complex quantitative traits relying on the cooperation of multiple loci in the genome, the use of molecular markers is essential to unravel the genetic base of phenotypic variability. The combination of marker technology and NGS allowed the detection of a huge number of DNA markers within a short time frame. The discovery of thousands of genetic markers across the whole genomes resulted largely advantageous either for the study of the genetic variability in wild populations, landraces, and cultivar collections or for the rapid genotyping of hundreds of individuals in a mapping cross focused on the identification of quantitative trait loci (QTLs). Largescale SNP datasets from biparental and multi-parental crossing populations (Borrill et al., 2018), and association panels are now free to access web resources available at CerealsDB (Wilkinson et al., 2016) and Ensembl Plants (Bolser et al., 2016). Genome-Wide Association Study has evolved promptly in the exploitation of natural variability in plant collections picking into the genome the polymorphic sequences responsible for variation in the trait of interest. Other sources of genetic variability, such as induced mutagenesis and TILLING, have also been benefited by NGS: exome capture has been used to sequence the genomes of thousands of individuals of mutagenized plant collections providing enormous advantages to the research of the genotypes of interest (Krasileva et al., 2017; Mo et al., 2018). Similarly, a multitude of RNA-Seq datasets has being generated and made available on the web platform expVIP (Ramírez-González et al., 2018;^1^) supporting the elucidation of key regulatory pathways and genes involved in different traits of interest (Figure 4).

Once identified genetic determinants, a main objective of functional genomics is the functional annotation. In this context, reverse genetic tools such as TILLING and Eco-TILLING remain efficient and competitive strategies to validate gene function and generate new genetic variability (McCallum et al., 2000; Comai et al., 2004). In Eco-TILLING, the coexistence of multiple polymorphisms across the plant genomes can allow the identification of plant haplotypes carrying meant polymorphisms at any locus involved in quantitative traits. Expression data are also useful to narrow down candidate genes identified through conventional QTL mapping or functional genomic approaches using a natural and induced variation. Classical transgenic approaches such as gene silencing and RNA-interference, have played a key role in functional genomics overall meant for polyploid organisms, such as wheat, where more than one gene copy should be switched off for perceptible phenotypes (Kumar et al., 2020).

## Searching for High Ax and We-Ax Content Qtls, Meta-Qtls, and Molecular Markers

Among cereals, AX has been mainly investigated in wheat. The wide network of metabolic pathways involved in AX modeling makes it difficult to target key enzymes to tune their structure and increase their amount. At now, the main work has been done in the exploitation of natural variability in AX content and the identification of related QTLs. Biparental population and large wheat collections have been phenotyped: extensive screening of the extract viscosity, mainly associated with WE-AX content, along with the A/X ratio, the amount of WE-AX and total AX, has been associated with a multitude of genetic loci spread throughout the wheat genome (Marcotuli et al., 2020). Combining the data of extract viscosity from five different wheat populations, Quraishi et al. (2009) optimized the work of different groups through the theorization of a “meta-QTL” including three loci on the chromosomes 1BL, 3D, and 6B. However, several reports analyzing large collections of wheat (inbred, diversity collections) identified many QTLs that mostly account for relatively little variation in AX content (lower than 15.2%). Most of these studies identified a QTL on chromosome 1BL as the most highly associated with the WE-AX content or WE-AX-related properties. At now, the coincidence of various QTLs on this chromosome has not yet been established: Yang et al. (2016) showed that the 1BL QTL, identified in a collection of 240 inbred lines, derived from the 1BL/1RS translocation from rye, known to be absent in modern wheats. More recently, an effective KASP marker for the 1BL QTL has been validated by Lovegrove et al. (2020). Here, two high AX content cultivars Yumai 34 (with high value in WE-AX; Tremmel-Bede et al., 2017) and Valoris, have been intercrossed and crossed with the other two cultivars. The four crosses revealed two major QTLs located on chromosome 1BL from Yumai-34 and chromosome 6B from Valoris. Similarly, Ibba et al. (2021) developed and validated four KASP markers for the 1BL QTL identified through a GWAS study across a collection of 175 wheat lines. Inquiring of the genomic regions surrounded by the associated markers identified a candidate gene involved in AX synthesis. Notably, while most of the research has been conducted on wholegrain material, these last two works focused on white flour.

The discovery of key genes for the manipulation of AX in cereals is challenging also due to the presence of multiple isoforms of the biosynthetic enzymes. In the studies of the gene function, pleiotropic effects of the not-targeted isoforms need to be considered (Bulone et al., 2019). In this regard, omics studies run both on the genomic and transcriptomic scale can help to clarify the role played by different isoenzymes.

## β-Glucan Genetic Variability: a Source to Boost β-Glucan Content and Viscosity and to Fine-Tune Its Structure

The concentrations of βG and DP3:DP4 ratios are significantly influenced by the genotype (Cory et al., 2017). In this regard, βG content and structure are complex traits controlled by a multitude of loci positioned on different chromosomes of the genomes. Major QTLs located on the chromosomes 1H, 2H, 5H and minor loci localized on 3H, 4H, and 6H have been identified (Molina-Cano et al., 2007; Szűcs et al., 2009; Pauli et al., 2014; Shu and Rasmussen, 2014). The QTL on chromosome 7H, localized in the centromeric region, is widely reported and accounts for up to 39% of variation βG content (Li et al., 2008a); an extra QTL located on the distal part of the same chromosome explained a 12.5% of variation; 1H and 5H impacted for the 7–15%. By the synteny with the QTLs identified in barley, key genes involved in βG synthesis were isolated in the rice genome, the first to be annotated among cereals. Many genes belonging to the two major families involved in βG synthesis, *CslF*, and *CslH*, have been isolated in genomic regions concerned by βG associated QTLs. A QTL on chromosome 2H in barley was associated with a cluster of five *Csl*F and one *CslH* gene: *CslF3*, *CslF4*, *CslF8*, *CslF10, CslF12*, and *CslH1*, while in rice a similar cluster of six *OsCslF* genes and two truncated *OsCslF* pseudogenes was detected on rice chromosome 7*; CslF6* was also mapped in the region of the major QTL on chromosome 7H; *CslF9* and a *CslH* isoform were associated to a QTL located on the 1H chromosome (Houston et al., 2014). Single-marker analyses suggested that the genetic control of β-glucan amount and DP3:DP4 ratio was linked to distinct chromosomal regions in the barley genome (chromosome 7H and 1H) (Cory et al., 2017).

In tetraploid wheat, QTLs have been identified on chromosomes 1A, 2A, 2B, 5B, and 7A (Marcotuli et al., 2016) which are associated with genes involved in starch, AX, and fructose metabolism, suggesting a significant role of carbon partitioning in the control of βG content, such as: a starch synthase SSIIa and an isoamylase essential in the elongation and modeling of amylopectin chains; a (1,4)-β-xylan endohydrolase involved in the definition of AX fine structure; a fructan 1-exohydrolase (1-FEH), which is involved in the hydrolysis of fructans have been located in the identified QTLs. Carbon partitioning is known to affect βG content in cereals. Diverse reports in barley, bread, and durum wheat have discussed pleiotropic effects on βG and AX content in starch mutants (Morell et al., 2003; Botticella et al., 2016, 2018). A GWAS study, performed in barley, reported a common QTL regulating amylose, amylopectin, and βG concentration identified in an operon-like structure on chromosome 7H (Shu and Rasmussen, 2014).

The investigation of the main regulators of the relationships between the metabolisms of the three DFs should be considered in future cereal research as an optimized approach to boost DFs content.

### Candidate Genes in β-Glucan Manipulation

The key role of some *CslF* genes has been validated in barley as well as in wheat and rice, by classical gene function studies: a mutation on the *HvCslF6* gene generated plants with a complete lack of βGs in the grain (Tonooka et al., 2009; Taketa et al., 2012); whereas the overexpression of *HvCslF6*, under the control of an endosperm-specific promoter, nearly doubled the content of βGs in the grain (Burton et al., 2011). In wheat transgenic plants with suppressed *TaCslF6*, βG content was decreased by 30–52% (Nemeth et al., 2010). In barley, the overexpression of *CslF4* increased grain βG content by 50% (Burton et al., 2011); differently, the transgenic expression of *CslF9* resulted ineffectively.

As regards the *β*-glucan structure, *CslF4* overexpression was associated with an increase in DP3:DP4 ratio (from 2.8:1 to 3.1:1), while the downregulation of the *CslF6* gene in the same genetic background decreased the DP3:DP4 ratio to 2.1:1.15. Silencing of endogenous *CslF6* in bread wheat did not affect the DP3:DP4 ratio but reduced the molecular weight of (1,3;1,4)-*β*-glucan (Nemeth et al., 2010). Additional candidate genes influencing the synthesis of βGs are *HvGlb1* and *HvGlb2* encoding (1,3;1,4)-β-d-glucan endohydrolase isoenzyme EI and EII and co-localized with significant QTLs at the pericentromeric region of 1HL (Han et al., 1995) and the distal end of 7HL, respectively (Li et al., 2008a; Houston et al., 2014). Although the essential role of *CslF6* in βG synthesis has been ascertained, the attempts to associate variation in βG content to polymorphisms in the gene sequence have been unsuccessful (Wong et al., 2015; Garcia-Gimenez et al., 2019). In a panel of 1,336 barley accessions showing a wide variation in βG content, the high conservation in the gene sequence was ascribed to the essential role played by CslF6 in grain vitality; further, the few detected polymorphisms did not explain any variation in βG content. Crossing between parents with extreme values in βG content did not detect stable molecular markers for *CslF6* across different environments and different cultivars (Cory et al., 2012, 2017; Wong et al., 2015). The comparison of gene expression of *CslF6* and other key genes involved in βG synthesis among different cultivars highlighted, in such case, different levels between high and low βG content barley genotypes; however, the mechanism and the genomic regions accounting for these differences are still under investigations (Wong et al., 2015). Different studies suggested a key role for the *CslH* gene family coding for (1,3;1,4)-β-endoglucanases, two isoenzymes co-localized with major QTLs at the pericentromeric region of 1HL (Han et al., 1995) and the distal end of 7HL (Li et al., 2008a; Houston et al., 2014). These genes were found differentially expressed between high and low βG content cultivars; β-endoglucanases could play a role in βG remodeling and be essential to provide a source of glucose from βG degradation in germinating seeds. It is generally assumed that other unknown proteins or regulatory factors are likely to be involved in the determination of βG content in seeds.

To enhance βG content in wheat, different groups tried to introgress key genes from barley through the creation of sets of interspecific addition/substitution lines carrying the chromosome with major QTLs (Danilova et al., 2019; Colasuonno et al., 2020). This approach was unable to balance barley βG content in wheat, supporting the hypothesis that multiple QTLs cooperate in the control of βG content, thus multiple key sequences need to be identified and joined to define a complete haplotype.

## Making Cereal Starch “Resistant”

High amylose genotypes have been produced in all the major cereals (Supplementary Table 2; Vineyard et al., 1958; Yano et al., 1985; Fujita et al., 1999; Regina et al., 2006; Botticella et al., 2011, 2018; Sestili et al., 2015). Strategies to manipulate the amylose/amylopectin ratio have pointed to target key enzymes involved in starch biosynthesis; two approaches have mainly been exploited: the overexpression of amylose synthesizing enzyme, GBSSI, and the silencing of enzymes involved in elongation and branching of amylopectin chains, namely SS and SBE. The first strategy increased the amount of GBSSI protein but did not increase the amylose content in durum wheat grain (Sestili et al., 2012). In contrast, Itoh et al. (2003) introduced a *GBSSI* transgene into a waxy rice mutant, obtaining transgenic lines with varying levels of amylose content up to 45%. In rice, amylose content ranges from 0 to ∼30% depending on the presence of different *Waxy* alleles, with *Wx*^*a*^ (high AC—more than 20%) and *Wx*^*b*^ (intermediate AC- 14 to ∼18%) being the major alleles found in the indica and japonica varieties, respectively (Teng et al., 2012). *Wx*^*b*^ harbors a mutation at the first nucleotide of intron 1 leading to a low expression of the gene; Crofts et al. (2018) reported that the lower amount of amylose in the genotypes harboring *Wx^*b*^* allele is due to the lower percentage of extra-long amylopectin chains synthesized by the less active GBSSI enzyme.

Although each enzyme showed to affect starch structures, the isoforms able to majorly advantage amylose content were identified in SS class II isoform a (SSIIa) and SBE class II (Wang et al., 2017). Elimination of SSIIa in cereals promotes amylose content from low to a medium level according to different species; moderate increase in amylose was found for maize *sugary2* (*su2*) and wheat *SSIIa* mutant (40–50%) (Yamamori et al., 2000; Zhang et al., 2004; Sestili et al., 2010a; Botticella et al., 2016). In barley *sex6* mutants, amylose increased up to 50% (Morell et al., 2003; Sparla et al., 2014). All these genotypes possess natural or induced mutations identified in wild/landraces collections and TILLING populations (Sestili et al., 2014): the selection of null SSIIa genotypes has been advantaged by the feasibility to visualize starch synthase by simple electrophoresis assays. Recently, the combination of SNPs, responsible for changes in key amino acids, in the three genes *GBSSI*, *SSIIa*, and *SSIIIa* was associated with a RS content of 8% (26% in amylose) in rice (Gurunathan et al., 2019). This finding highlights the potential of missense mutations to fine-tune traits of interest.

Superior outcomes arise from the suppression of SBEII activity. SBEII defective genotypes have been derived essentially by the screening of mutant collections or by transgenic silencing technology. Among cereals, SBEIIb isoform is the major in maize and rice, while in wheat and barley the most abundant is the “a” isoform (SBEIIa) (Regina et al., 2005). This difference well correlates with the diverse results in the modification of starch composition consequent to the suppression of the “a” or “b” isoforms among the different species. Inactivation of SBEIIb, which is 50 times more abundant than “a” in maize, promotes amylose content from 25–30 to 61–67% in a*mylose-extender* (*ae*) mutants (Li et al., 2008b). In rice, SBEIIb/SBEIIa ratio is much lower (5:1) thus reducing the extent of the rise in amylose to 15% in the *ae* mutant (Nishi et al., 2001). Another *ae*-like mutant *Goami2*, from a *japonica* rice variety, showed about a twofold increase in amylose (Kang et al., 2003) but it has been imputed to the incidence of a further mutation not yet discovered.

On the other hand, SBEIIa is the most significant isoform in wheat and barley (Botticella et al., 2012): removal of its activity by gene silencing raised amylose content to 75% in transgenic bread and durum wheat (25–30% in the control) (Regina et al., 2006; Sestili et al., 2010b); the outcome was different in barley, where a substantial increase in amylose (65%) was obtained only in lines where the expression of both SBEII isoforms resulted decreased by 80% (Regina et al., 2010). The two isoenzymes in barley share the branching activity with distinctive patterns of chain length being transferred by each other in the amylopectin backbone. High amylose wheats have also successfully been derived by the use of TILLING platforms both in bread and durum wheat (Botticella et al., 2011, 2018; Hazard et al., 2012; Slade et al., 2012; Sestili et al., 2015): amylose content increased from 26–33% of wild type to 55–70% in the two *SBEIIa* mutants derived from bread wheat cultivars Express and Cadenza, respectively; similarly, the silencing of *SBEIIa* genes in durum wheat cultivars, Kronos and Svevo, raised the amount of amylose from 24–30 to 47–52%, respectively. In all these lines the increase in amylose was associated with a strong increase in the RS fraction: up to 11–12% and 7% in bread and durum wheat mutants, respectively, compared to less than 1% detectable in wild type sib lines.

A further improvement was achieved by targeting more SBE isoforms in the same plant. In barley, an “amylose only” genotype was produced through the simultaneous suppression of both SBEII and SBEI isoforms through RNA interference (Carciofi et al., 2012).

Crossing between mutant lines derived from two mutagenized wheat populations, one obtained by treatment with physical agents in the cv Chara and the other by treatment with chemical agents in the cv Sunstate, confirmed a major impact for SBEIIa enzyme but also that, double mutation of the two isoforms, SBEIIa and SBEIIb, in wheat is cooperative for the increase in amylose and RS. RS raised to 16.6% of whole flour in the line with 84% in amylose content (Regina et al., 2015). Similarly, Li et al. (2019) produced bread wheat with 93.3% in amylose content (36.7% in the sib line), by combining null mutations in *SBEIIa* and *SBEIIb* genes derived by chemical mutagenesis.

Combining predicted defective allelic variants of SBEIIa and SBEIIb isoforms did not highly enhance the amylose in durum wheat cultivar Kronos (Hazard et al., 2014). The reasons for this discrepancy still need to be investigated. Recently, CRISPR-Cas9 technology was used to produce high amylose rice, raising RS up to 9% in SBEIIb defective mutant lines (Sun et al., 2017). The same approach has been successfully adopted by Li et al. (2021) to target *SBEIIa* genes in both winter and spring wheat varieties, generating transgene-free wheats with high-amylose (up to 69.7% of total starch) and high RS (up to 15%).

## Future Perspectives

Considering the notions here reviewed, much work could be done to produce high fiber cereals by adopting biotechnological tools. Generally, the digestibility of polysaccharides depends on the structure, molecular weight, the nature of glycosidic bonds, and the grade of crosslinking among glucans. The presence of side chains and the coexistence of more types of linkages among monomers increase the solubility of polymers that results in higher accessibility to the solvent and hydrolytic enzymes produced by microflora. For starch, where glucose moieties are linked by α-type bonds, the linearity and molecular weight positively correlate with a decrease in the susceptibility to the human’s amylases thus increasing the fraction (RS) available for fermentation by microbiota.

One challenge to be accomplished remains the identification of the main genetic determinants of DFs’ fine structure.

As regards AX, the manipulation of arabinose content can positively affect the solubility of the polymer; for this purpose, targeting enzymes involved in both synthesis and transport of Golgi-derived UDP-sugar precursors can help to increase arabinose side chains in the AX structure. However, some arabinose residues may represent substrates for esterification by ferulic acid that reduces its solubility. Consequently, it would be of interest to distinguish isoenzymes responsible for the incorporation of arabinose residues at different positions in the polysaccharide chain. To this aim, primarily reverse genetics tools (i.e., mutagenesis, genome editing, transgenesis) will be essential to identify the key isoenzymes. Knowledge of gene sequences and the elucidation of the pangenome of cereal species (Della Coletta et al., 2021) coupled with functional investigations will facilitate the selection of the best candidate genes to be genetically manipulated. Therefore, further advances can derive by QTLs identification accelerated by NGS-based molecular markers. Similarly, βGs solubility is mainly ascribed to DP3/DP4 ratio; here the elucidation of the role played by specific members of CslF and CslH along with other unknown regulators can be pursued by functional omics studies based on the comparison of high and low content genotypes; moreover, the investigation of the catalytic properties of single isoenzymes by *in vitro* assays could give an important contribution. Also, regards RS, many opportunities can arise by a better understanding of mechanisms regulating starch fine structure; indeed, molecular weight and branching patterns are finely regulated by a complex of enzymes where the exact role played by each member in the diverse cereals has not yet completely pointed out. In this sense, the targeting of specific enzymes coupled with a detailed investigation of the resultant changes in the fine structure will provide novel opportunities for starch applications in the food industry.

A further issue to face regards the mitigation of side effects associated with high DF content genotypes. Indeed, it has been ascertained that the manipulation of their biosynthetic pathways can affect the metabolic networks causing carbon re-allocation among the compounds in the seed (Morell et al., 2003; Botticella et al., 2018). Occasionally, high amylose starch genotypes display a decreased total starch content with a yield penalty. For this purpose, one strategy can be to introgress yield-associated alleles in such genotypes as well as crossing them with high yield cultivars. Furtherly, for polyploids species such as wheat, it is of interest to evaluate the phenotypes of partial mutants, having mutations in some homoeologous, to find a good balance between improved quality and acceptable yield. The other two traits to be studied in high DF genotypes are the resistance to biotic stress and seed germination rate, since changes in the cell wall structure, as well as the reduction in starch digestibility, can impact both physiological processes; for instance, changes in starch and βGs, essential nutriments for seed development, can impact on seed germination rate.

A further side effect in starch mutants is the change in hardness: in wheat, hardness is an important trait for the technological and qualitative properties of flour. The increase in hardness of high amylose genotypes can have negative impacts in milling with a reduced flour yield and a high percentage of damaged starch (Botticella et al., 2018).

To address this issue, the introgression of the high amylose character in soft cultivars could be ideal for the creation of high RS lines.

Several investigations have found an association between the three DFs here discussed: AXs and b-glucan were increased in high and low amylose lines in several species (Morell et al., 2003; Botticella et al., 2018) but still few studies have investigated this relationship at the metabolic level and the key regulators are still unknown. For this purpose, new knowledge will permit the development of new crop improvement programs aimed to increase the content of more than one type of DFs in the same plant.

Considering the urgency to fight malnutrition through a global approach, a further objective is to combine multiple healthy traits in the same genotype; increasing the content of essential micronutrients such as microelements or vitamin precursors in high fiber genotypes will produce super crops aimed at satisfying multiple nutrition needs at the same time. At this purpose, genome editing technologies, able to target multiple genes simultaneously, may greatly accelerate breeding programs; these new approaches accomplished to the advent of “speed breeding” (Watson et al., 2018) will permit to respond rapidly and efficiently to the new challenges, such as the climate changes, the necessity to increase crop yield and to realize new plants more nutritious and healthy for humans.

## Conclusion

NGS and the novel biotechnological tools have enormously accelerated the elucidation of nutrient metabolism and the identification of key loci responsible for their accumulation in the plant, representing one of the best approaches to gain biofortification of staple crops. Genotyping of hundreds of individuals has become feasible in a short time frame, allowing the identification of multiple loci determining complex traits and, overall, increasing the possibility of genetic variability exploitation in complex crops such as cereals. Among DFs, βGs and AXs are cell wall polysaccharides more concentrated in the outer layers of the seed, while RS is essentially associated with high amylose starches only found in the endosperm. If candidate genes for RS content have been identified and successfully targeted to increase its content, both for AXs and βGs this is far longer to be achieved also due to the high number of members belonging to the large family of the biosynthetic enzymes. Nonetheless, the high potential of modern omics and biotechnological tools is accelerating the identifications of major QTLs and key biosynthetic enzymes thus paving the way for the development of novel high fiber genotypes in major cereals.

## Author Contributions

All authors listed have made a substantial, direct and intellectual contribution to the work, and approved it for publication.

## Conflict of Interest

The authors declare that the research was conducted in the absence of any commercial or financial relationships that could be construed as a potential conflict of interest.

## Publisher’s Note

All claims expressed in this article are solely those of the authors and do not necessarily represent those of their affiliated organizations, or those of the publisher, the editors and the reviewers. Any product that may be evaluated in this article, or claim that may be made by its manufacturer, is not guaranteed or endorsed by the publisher.

## Footnotes

1
www.wheat-expression.com/

## Supplementary Material

Supplementary Figure 1 Dietary fibers across seed layers in major cereals. The figure reports the major dietary fibers and their localization in the different kernel layers. AX, arabinoxylans; βG, βglucans; RS, resistant starch.

Supplementary Table 1 Amount of DFs in different seed tissues of major cereals.

Supplementary Table 2 High Resistant starch genotypes in major cereals.
