# Supplementary figures and images for: The Triple Jags of Dietary Fibers in Cereals: How Biotechnology Is Longing for High Fiber Grains
Source: Front Plant Sci. 2021 Sep 14;12:745579. doi: 10.3389/fpls.2021.745579 (PMC8477015; doi:10.3389/fpls.2021.745579)

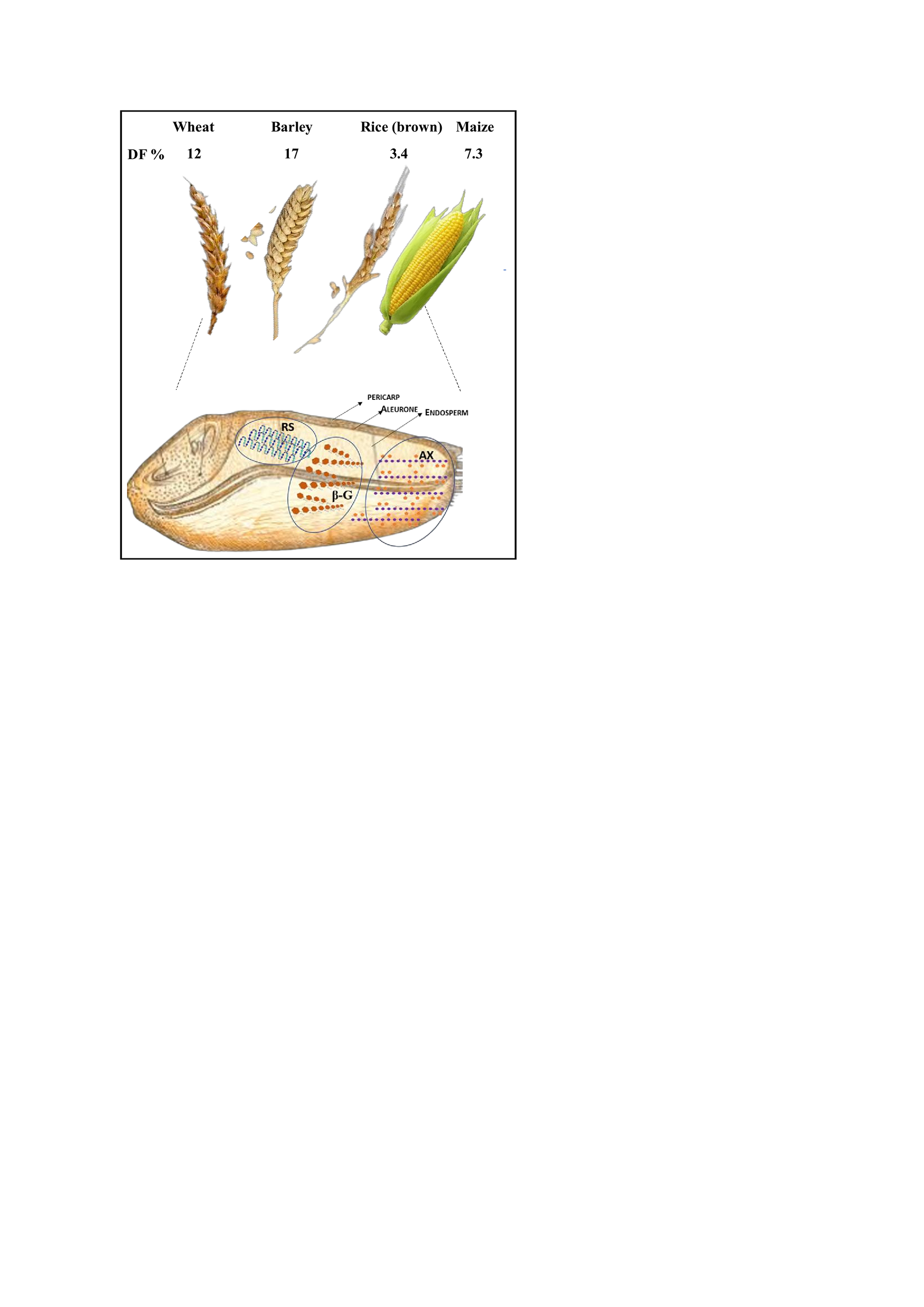

Supplement: Supplementary Figure 1 — Dietary fibers across seed layers in major cereals. The figure reports the major dietary fibers and their localization in the different kernel layers. AX, arabinoxylans; βG, βglucans; RS, resistant starch. [file Image_1.TIF]
