# Supplementary material for: The Triple Jags of Dietary Fibers in Cereals: How Biotechnology Is Longing for High Fiber Grains
Source: Front Plant Sci. 2021 Sep 14;12:745579. doi: 10.3389/fpls.2021.745579 (PMC8477015; doi:10.3389/fpls.2021.745579)
Supplement: Supplementary Table 1 — Amount of DFs in different seed tissues of major cereals. [file Table_1.docx]

**Supplementary Table 1. Amount of DFs in the different seed tissues of major cereals**.

|  | **Barley** | | | | **Wheat** | | | | **Rice** | | | | **Maize** | | | |
| --- | --- | --- | --- | --- | --- | --- | --- | --- | --- | --- | --- | --- | --- | --- | --- | --- |
|  | **Whole grain** | **P** | **A** | **E** | **Whole grain** | **P** | **A** | **E** | **Whole grain** | **P** | **A** | **E** | **Whole grain** | **P** | **A** | **E** |
| **TOT-AX (%)** | 4.69 | 40.1 | 25.55 | 1.64 | 5.9 | 46.2 | 20.8 | 1.6 | 8.99 | 26.7^9^ | 13.2^9^ | na | 5.1-6.8^10^ | na | na | na |
| **WE-AX (%)** | 22.6 | 0.9 | 1.34 | Na | 0.3-0.9^10^ | Na | na | na | 0.15-1.2 | Na | na | na | 0.28 | na | na | na |
| **β-glucan (%)** | 2-20^10^ | na | na | Na | 0.48^1^ | Na | na | na | <2^2^ | Na | na | na | <2^2^ | 6.6^3^ | na | na |
| **RS** | 0.13-0.37^4^ | na | na | Na | 0.2-0.83^5,6^ | Na | na | na | 0.35-2.57^7^ | Na | na | na | 0.67^8^ | na | na | na |

P: pericarp, A: aleurone, E: endosperm; na: not available; ^1^Havrlentov and Kraic, 2006; ^2^Marcotuli et al., 2019;^3^Yoshida et al., 2014. ^4^Abdel-Aal and Choo, 2014. ^5^Botticella et al., 2018; ^6^Slade et al., 2012; ^7^Kumar et al., 2018. ^8^McCleary et al., 2002; ^9^Wang et al., 2016; ^10^Collins et al., 2010.

**References**

Abdel-Aal, E.-S. M., and Choo, T.-M. (2014). Differences in compositional properties of a hulless barley cultivar grown in 23 environments in eastern Canada. *Can. J. Plant Sci.* 94, 807–815.

Collins, H. M., Burton, R. A., Topping, D. L., Liao, M.-L., Bacic, A., and Fincher, G. B. (2010). Variability in fine structures of noncellulosic cell wall polysaccharides from cereal grains: potential importance in human health and nutrition. *Cereal Chem.* 87, 272–282.

Havrlentov, M. and Kraic, J. (2006). Content of β-D-glucan in cereal grains. *J. Food Nutr. Res.* 45, 97–103.

Kumar, A., Sahoo, U., Baisakha, B., Okpani, O. A., Ngangkham, U., Parameswaran, C., et al. (2018). Resistant starch could be decisive in determining the glycemic index of rice cultivars. *J. Cereal* *Sci.* 79, 348–353.

Marcotuli, I., Colasuonno, P., Cutillo, S., Simeone, R., Blanco, A., and Gadaleta, A. (2019). β-glucan content in a panel of Triticum and Aegilops genotypes. *Genet. Resour. Crop Evol.* 66, 897–907.

McCleary, B. V., McNally, M., and Rossiter, P. (2002). Measurement of resistant starch by enzymatic digestion in starch and selected plant materials: collaborative study. *J. AOAC Int.* 85, 1103–1111.

Wang, J., Suo, G., de Wit, M., Boom, R. M., and Schutyser, M. A. (2016). Dietary fibre enrichment from defatted rice bran by dry fractionation. *J. Food Eng.*186, 50–57.

Wang, Y. J., White, P. J., Pollak, L. M., and Jane, J. (1993). Characterization of starch structures of 17 maize endosperm mutant genotypes with Oh43 inbred line background. *Cereal Chem.* 70, 171.

Yoshida, T., Honda, Y., Tsujimoto, T., Uyama, H., and Azuma, J. (2014). Selective isolation of β-glucan from corn pericarp hemicelluloses by affinity chromatography on cellulose column. *Carbohydr. Polym.* 111, 538–542.
