# Supplementary material for: The Triple Jags of Dietary Fibers in Cereals: How Biotechnology Is Longing for High Fiber Grains
Source: Front Plant Sci. 2021 Sep 14;12:745579. doi: 10.3389/fpls.2021.745579 (PMC8477015; doi:10.3389/fpls.2021.745579)
Supplement: Supplementary Table 2 — High Resistant starch genotypes in major cereals. [file Table_2.docx]

|  | Varietal background | Name | How | Target | AC %*  RS %* | Reference |
| --- | --- | --- | --- | --- | --- | --- |
| Rice  (Oryza sativa) | Taichung 65(J*-Wx^b^)  Kinmaze (J**-Wx^b^) | #1403 | Mutagenesis | *SBEI/SBEIIb* | 51.7 (21.1)  35.1 (0.2) | Miura et al., 2021 |
|  | Ilpumbyeo (J**) | *Goami2*  *Suweon 464* | Mutagenesis | - | 34 (18)  - | Kang et al., 2003 |
|  | Nipponbare (J**) | *e1* | Mutant | *SSIIIa* | 30.7 (21.2) | Fujita et al., 2007 |
|  | Kinmaze (J**) | - | Mutant | *-* | 29-34 (17)  - | Yano et al., 1985 |
|  | Kinmaze (J**) | EM_10_ | Mutant | *SBEIIb* | 28.1 (21.6)  11.6 (0.2) | Nishi et al., 2001 |
|  | - | - | Mutant | *SS1/SSIIIa* | 33 (21)  - | Fujita et al., 2011 |
|  | Nipponbare | - | hp-RNA silencing | *SBEIIb* | 41.2 (19.6)  4.8 (-) | Butardo et al., 2011 |
|  | (I**-Wx^a^) | HA-1 HA-2 | RNA-i | *SBEI/SBEIIb* | 64.8 (27.2)  14.6 (-) | Zhu et al., 2012 |
|  | Kitaake (J**) | - | CRISPR-Cas9 | *SBEIIb* | 25 (15)  9.8 (0.1) | Sun et al., 2017 |
|  | Kinmaze (J**) | - | Mutant | *SSIIIa/SBEIIb* | 45.1 (21)  - | Asai et al., 2014 |
| Mais  (Zea mays) | H99ae, OH43ae, B89ae, B84ae | amylose extender (ae) | Mutant | *SBEIIb* | 61-68 8 (-)  18.6-20.9 (-) | Li et al., 2008*b* |
|  | GUAT209:S13 × (OH43ae × H99ae)] |  | Mutant | *SBEIIb-SBEI* | 68.9-88.2 (-)  39.4–43.2 (-) | Li et al., 2008*b* |
|  | Standard line x Mutator line | sugary 2 (su2) | Mu transposable element | *SSIIa* | 38-40 (26)  - | Zhang et al., 2004 |
|  | Oh43 Inbred Line | dull1 (du1) | Mutant | *SSII* | 31.4 (27.8)  - | Wang et al., 1993 |
|  | Oh43 Inbred Line | sugary 1 (su1) | Mutant | *ISA1* | 37.4 (27.8)  - | Wang et al., 1993 |
|  | Oh43 Inbred Line | ae du1 | Mutant | *SBEIIb-SSII* | 57.8 (27.8)  - | Wang et al., 1993 |
|  | Oh43 Inbred Line | du1 su1 | Mutant | *SSII-ISA1* | 39.9 (27.8)  - | Wang et al., 1993 |
| Barley  (*Hordeum vulgare*) | Glacier | *amo1* Glacier AC38 | Mutant | *amo1* | 37.4 (25.7)  - | Banks et al., 1971; Yoshimoto et al., 2000 |
|  | Himalaya | *Sex6* Himalaya 292 | Mutagenesis | *SSIIa* | 59 (32)  1.9 (-) | Morell et al., 2003 |
|  | GlacierAC38/Himalaya292 | *Sex6 /amo1* | Mutagenesis | *SSIIa/amo1* | 61 (32)  3.4 (1.2) | Li et al., 2011 |
|  | Golden Promise | - | RNA-i | *SBEIIa/SBEIIb* | 69.4 (31.4)  6.3 (0.2) | Regina et al., 2010 |
|  | Golden Promise | - | RNA-i | *SBEIIa/SBEIIb/SBEI* | 99 (29)  90 (-) | Carciofi et al., 2012 |
| Bread Wheat  (*Triticum aestivum*) | Chousen30  Kanto79  Turkey116 | SGP1 null | Natural mutant | *SSIIa* | 37.3 (29.6)  3.64 (0.02) | Yamamori et al., 2000; Yamamori et al., 2006 |
|  | Cadenza | Cad SSIIa* | Mutagenesis | *SSIIa* | 45.7 (34.0)  1.4 (0.2) | Botticella et al., 2018 |
|  | Jagger | Jag-ssiia-∆ABD | TILLING | *SSIIa* | 35.7 (31.1)  2.21 (1.0) | Schoen et al., 2021 |
|  | Express | - | TILLING | *SBEIIa* | 55.7 (22.9)  6.5 (0.8) | Slade et al., 2012 |
|  | Cadenza | Cad-SBEIIa | TILLING | *SBEIIa* | 78.7(34.0)  7.2 (0.2) | Botticella et al., 2011; Botticella et al., 2018 |
|  | Zhengmai7698 (winter) | ZM Mutant | CRISPR/CAS9 | *SBEIIa* | 65.4 (30.6)  6.6 (1.2) | Li et al., 2020 |
|  | Bobwhite (spring) | Bobwhite Mutant |  |  | 69.7 (30.6)  8.7 (1.8) |  |
|  | Express/Chara | A1B2nD2 | Physical Mutagenesis /TILLING | *SBEIIa/SBEIIb* | 84.4 (29.6)  16.6 (0.9) | Regina et al., 2015 |
|  | Express | - | TILLING | *SBEIIa/SBEIIb* | 63.8 (36.7)  - | Li et al., 2019 |
| Durum Wheat  (*Triticum durum*) | Mountrail | - | (Natural mutants/EMS mutagenesis) | *SSIIa* | 44.3 (28.7)  - | Hogg et al., 2013 |
|  | Svevo | - | Natural mutant | *SSIIa* | 46.0 (31.0)  3.0 (0.4) | Botticella et al., 2016 |
|  | Kronos | - | TILLING | *SBEIIa* | 47.4 (24.4)  6.2 (1.6) | Slade et al., 2012 |
|  | Kronos | - | TILLING | *SBEIIa* | 28 (23)  1 (0.4) | Hazard et al., 2012 |
|  | Svevo | - | TILLING | *SBEIIa* | 54.4 (26.9)  6.5 (0.8) | Sestili et al., 2015 |
|  | Svevo | MJ16-112 | RNAi | *SBEIIa* | 75.0 (24.5)  - | Sestili et al., 2010*b* |
|  | Ofanto | A428 |  |  | 56.4 (30.9)  - |  |
|  | Kronos | - | TILLING | *SBEIIa/SBEIIb* | 40.1 (26.6)  3.7 (0.5) | Hazard et al., 2014 |

*I, Indica; J, Japonica; SSIIa from japonica rice cultivars contains four amino acid replacements compared with indica rice cultivars and three of these are associated with significant reduction in japonica SSIIa activity ([Nakamura et al., 2005](https://www.frontiersin.org/articles/10.3389/fpls.2018.00645/full#B23))

**References**

Asai, H., Abe, N., Matsushima, R., Crofts, N., Oitome, N. F., Nakamura, Y., et al. (2014). Deficiencies in both starch synthase IIIa and branching enzyme IIb lead to a significant increase in amylose in SSIIa-inactive japonica rice seeds. *J. Exp. Bot.* 65, 5497–5507. doi:[10.1093/jxb/eru310](https://doi.org/10.1093/jxb/eru310).

Banks, W., Greenwood, C. T., and Walker, J. T. (1971). Studies on the starches of barley genotypes. A comparison of the starches from normal and high‐amylose barley. *Starch‐Stärke* 23, 12–15.

Butardo, V. M., Fitzgerald, M. A., Bird, A. R., Gidley, M. J., Flanagan, B. M., Larroque, O., et al. (2011). Impact of down-regulation of starch branching enzyme IIb in rice by artificial microRNA- and hairpin RNA-mediated RNA silencing. *J. Exp. Bot.* 62, 4927–4941. doi:[10.1093/jxb/err188](https://doi.org/10.1093/jxb/err188).

Fujita, N., Satoh, R., Hayashi, A., Kodama, M., Itoh, R., Aihara, S., et al. (2011). Starch biosynthesis in rice endosperm requires the presence of either starch synthase I or IIIa. *J. Exp. Bot.* 62, 4819–4831.

Fujita, N., Yoshida, M., Kondo, T., Saito, K., Utsumi, Y., Tokunaga, T., et al. (2007). Characterization of SSIIIa-deficient mutants of rice: the function of SSIIIa and pleiotropic effects by SSIIIa deficiency in the rice endosperm. *Plant Physiol.* 144, 2009–2023. doi:[10.1104/pp.107.102533](https://doi.org/10.1104/pp.107.102533).

Hogg, A. C., Gause, K., Hofer, P., Martin, J. M., Graybosch, R. A., Hansen, L. E., et al. (2013). Creation of a high-amylose durum wheat through mutagenesis of starch synthase II (SSIIa). *J. Cereal Sci.* 57, 377–383.

Li, Z., Li, D., Du, X., Wang, H., Larroque, O., Jenkins, C. L. D., et al. (2011). The barley amo1 locus is tightly linked to the starch synthase IIIa gene and negatively regulates expression of granule-bound starch synthetic genes. *J. Exp. Bot.* 62, 5217–5231. doi:[10.1093/jxb/err239](https://doi.org/10.1093/jxb/err239).

Miura, S., Koyama, N., Crofts, N., Hosaka, Y., Abe, M., and Fujita, N. (2021). Generation and Starch Characterization of Non-Transgenic BEI and BEIIb Double Mutant Rice (Oryza sativa) with Ultra-High Level of Resistant Starch. *Rice* 14, 1–16.

Nakamura, Y., Francisco, P. B., Hosaka, Y., Sato, A., Sawada, T., Kubo, A., et al. (2005). Essential amino acids of starch synthase IIa differentiate amylopectin structure and starch quality between japonica and indica rice varieties. *Plant Mol. Biol.* 58, 213–227.

Schoen, A., Joshi, A., Tiwari, V., Gill, B. S., and Rawat, N. (2021). Triple null mutations in starch synthase SSIIa gene homoeologs lead to high amylose and resistant starch in hexaploid wheat. *BMC Plant Biol.* 21,. 74. doi:[10.1186/s12870-020-02822-5](https://doi.org/10.1186/s12870-020-02822-5)

Yamamori, M., Kato, M., Yui, M., and Kawasaki, M. (2006). Resistant starch and starch pasting properties of a starch synthase IIa-deficient wheat with apparent high amylose. *Aust. J. Agr .Res.* 57, 531–535.

Yoshimoto, Y., Tashiro, J., Takenouchi, T., and Takeda, Y. (2000). Molecular Structure and Some Physicochemical Properties of High-Amylose Barley Starches. *Cereal Chem. J.* 77, 279–285. doi:[10.1094/CCHEM.2000.77.3.279](https://doi.org/10.1094/CCHEM.2000.77.3.279).

Zhu, L., Gu, M., Meng, X., Cheung, S. C. K., Yu, H., Huang, J., et al. (2012). High-amylose rice improves indices of animal health in normal and diabetic rats. *Plant* *Biotechnol.* *J.* 10, 353–362.
